# The Study of Misclassification Probability in Discriminant Model of Pattern Identification for Stroke

**DOI:** 10.1155/2016/1912897

**Published:** 2016-03-10

**Authors:** Mi Mi Ko, Honggie Kim

**Affiliations:** ^1^KM Fundamental Research Division, Korea Institute of Oriental Medicine, Daejeon 305-811, Republic of Korea; ^2^Department of Information and Statistics, Chungnam National University, Daejeon 305-764, Republic of Korea

## Abstract

*Background*. Pattern identification (PI) is the basic system for diagnosis of patients in traditional Korean medicine (TKM). The purpose of this study was to identify misclassification objects in discriminant model of PI for improving the classification accuracy of PI for stroke.* Methods. *The study included 3306 patients with stroke who were admitted to 15 TKM hospitals from June 2006 to December 2012. We derive the four kinds of measure (*D*, *R*, *S*, and *C* score) based on the pattern of the profile graphs according to classification types. The proposed measures are applied to the data to evaluate how well those detect misclassification objects.* Results.* In 10–20% of the filtered data, misclassification rate of *C* score was highest compared to those rates of other scores (42.60%, 41.15%, resp.). In 30% of the filtered data, misclassification rate of *R* score was highest compared to those rates of other scores (40.32%). And, in 40–90% of the filtered data, misclassification rate of *D* score was highest compared to those rates of other scores. Additionally, we can derive the same result of *C* score from multiple regression model with two independent variables.* Conclusions.* The results of this study should assist the development of diagnostic standards in TKM.

## 1. Introduction

Due to the development of modern medicine, the average lifespan for human beings is anticipated to rise beyond 85 years of age within the following 20 years [1]. In the meantime, since the rate of aging in South Korea is expected to surge up to 35.1% by 2050, ranking 2nd in the world close to Japan (37.7%), geriatric diseases and the health of the elderly have emerged as one of the most critical social problems of improving the quality of life in the future [2]. In particular, stroke is one of the representative geriatric diseases, along with dementia. Personal and social insecurities caused by the disease have continued to grow. In addition, stroke ranks as the top mortality risk to Koreans among the single diseases and contributes to more than 70% of the in-patients at traditional Korean medical hospitals [3, 4]. In traditional Korean medicine (TKM), specific or nonspecific symptoms of patients are diagnosed by observing, listening, asking, and feeling their pulse under the diagnostic system of pattern identification (PI) in order to determine the cause, nature, treatment method, and treatment drugs of a disease [5–7]. This PI diagnosis collects specific or nonspecific symptoms of patients and classifies them into one of the hundreds of symptom classes. It is the essential core technology forming the backbone of diagnosis and treatment in oriental medicine. However, the PI diagnosis holds limited objectivity and reproducibility due to the lack of standardized measurement indices, and objectification problems have always arisen with respect to personal deviations among TKM physicians based on their knowledge and experience [6–8].

As the necessity for the standardization of diagnostic systems has recently come to the fore, studies have been underway to objectify diagnosis.

In the study titled “Fundamental Study for the Standardization and Objectification of Pattern Identification in Traditional Korean Medicine for Stroke (SOPI-Stroke),” which was conducted over 9 years from 2005 to 2013, the Korea Institute of Oriental Medicine (KIOM) proposed a standardization plan for PI/syndrome differentiation of stroke, established stroke PI diagnostic indices, built a database system relating to TKM clinical technologies by setting up a clinical index database, and founded a scientific basis for stroke and PI by discovering stroke and PI biological indices, to which the latest research methods, such as OMICS, were applied. Studies were carried out to discover biological indices that could be helpful to stroke prevention by finding out what the stroke risk factors were [9–16].

Consequently, the purpose of this study was to identify misclassification objects in discriminant model of PI for improving the classification accuracy of PI for stroke patients. Although current TKM PI diagnostic tools for stroke were developed after several years of research and prepared for public release, the tools still need corrections and modifications in many aspects [17–19]. In this study, the key topics for discussion involve appropriate statistical methods to reduce the probability of diagnostic misclassification.

## 2. Methods

### 2.1. Subjects

The study included 3306 patients with stroke who were admitted to 15 oriental medical university hospitals from June 2006 to December 2012. Each patient provided informed consent to undergo procedures that were approved by the respective institutions' Institutional Review Boards (IRB). Informed consent of all the study patients was obtained after a thorough explanation of the details. We enrolled stroke patients for enrollment within 30 days of the onset of their symptoms, provided that their diagnosis was confirmed by an imaging diagnosis such as computerized tomography (CT) or magnetic resonance imaging (MRI). Patients with traumatic stroke such as subarachnoid, subdural, and epidural hemorrhage were excluded from the study.

### 2.2. Measured Variables

Each patient was seen by two experts at the same department within each site. All experts who were well trained in standard operation procedures (SOPs) were participating in this study. The experts had at least three years of clinical experiences with stroke after finishing regular college education about TKM for six years. The examination parameters were extracted from parts of a case report form (CRF) for the standardization of stroke diagnosis that had been developed by an expert committee organized by the KIOM [7, 11, 12].

#### 2.2.1. The Korean Standard PI for Stroke-3

PI process for differentiating stroke with four TKM types: the Fire-heat (FH) pattern, Dampness-phlegm (DP) pattern, Yin deficiency (YD) pattern, and Qi deficiency (QD) pattern [11, 12]. The FH pattern is characterized by any symptom of heat or fire that is contracted externally or engendered internally. The DP pattern is characterized by impeding Qi movement and its turbidity, heaviness, stickiness, and downward-flowing properties. The QD pattern is characterized by qi deficiency with diminished internal organ function, which is marked by shortness of breath, lassitude, listlessness, spontaneous sweating, a pale tongue, and a weak pulse. The YD pattern is characterized by yin deficiency with diminished moistening and the inability to restrain yang, which is usually manifested as fever [7, 9–13, 20]. The Korean Standard PI for Stroke-3 consists of 44 clinical indices and each clinical index belongs to its respective PI (Supplemental Table 1, in Supplementary Material available online at http://dx.doi.org/10.1155/2016/1912897).

### 2.3. Statistical Methods

After determining 12 different types of misclassification through discriminant analysis, we plotted it on the profile graphs according to types. And then we derive the four kinds of measure (*D*, *R*, *S*, and *C* score) based on the pattern analysis of the profile graphs. The proposed measures are applied to the stroke data to evaluate how well those detect misclassification objects.

#### 2.3.1. Types of Misclassification

**Figure 1:**
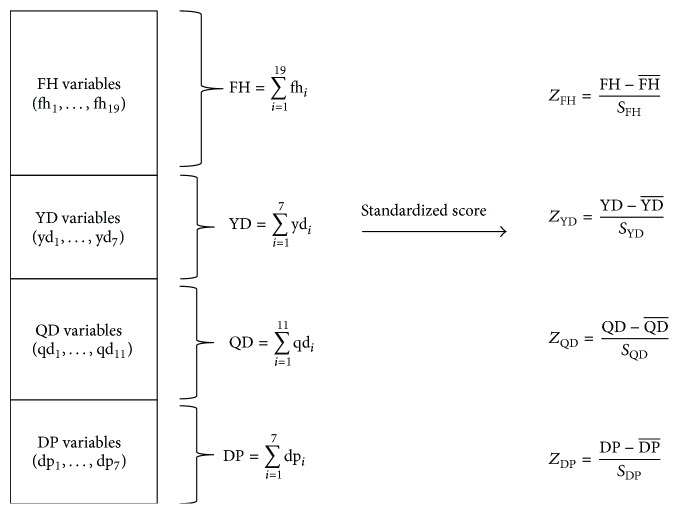
Process of grouping of explanatory variables and standardized scores generation. The mean and standard deviation of each upper-class variable were used to attain standardized scores, after which the misclassification types were analyzed. QD: Qi deficiency pattern; DP: Dampness-phlegm pattern; YD: Yin deficiency pattern; FH: Fire-heat pattern.

According to the results from the discriminant model classification, 2,209 patients posted correct classifications out of the total of 3,306 patients (66.82%) (Table 1). Out of the 3,306 patients, 1,097 were misclassified (33.2%) and the misclassification types are summarized in Table 2. To analyze the misclassification types, 44 clinical indices of the Korean Standard PI for Stroke-3 were grouped into four upper-class variables (QD, DP, YD, and FH pattern indices). In addition, the average and standard deviation of each upper-class variable was used to attain standardized scores, after which the misclassification types were analyzed (Figure 1).

#### 2.3.2. The Profile Graphs

**Figure 2:**
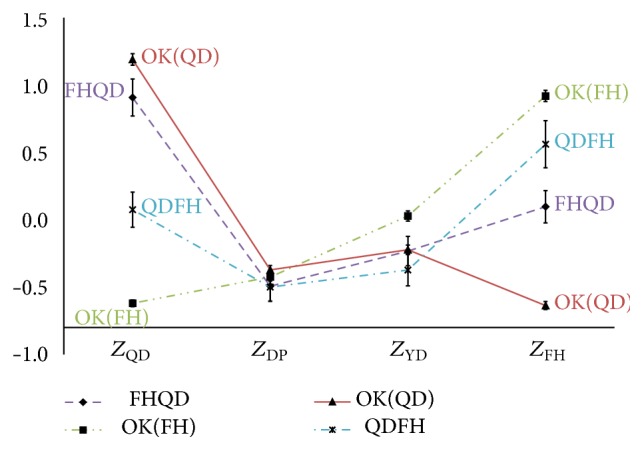
The profiles graphs of the FH and QD. *Z*_FH_: the standardized scores for upper-class variables according to Fire-heat pattern; *Z*_QD_: the standardized scores for upper-class variables according to Qi deficiency pattern; *Z*_DP_: the standardized scores for upper-class variables according to Dampness-phlegm pattern; *Z*_YD_: the standardized scores for upper-class variables according to Yin deficiency pattern; OK: the correct classification types.

**Figure 3 fig3:**
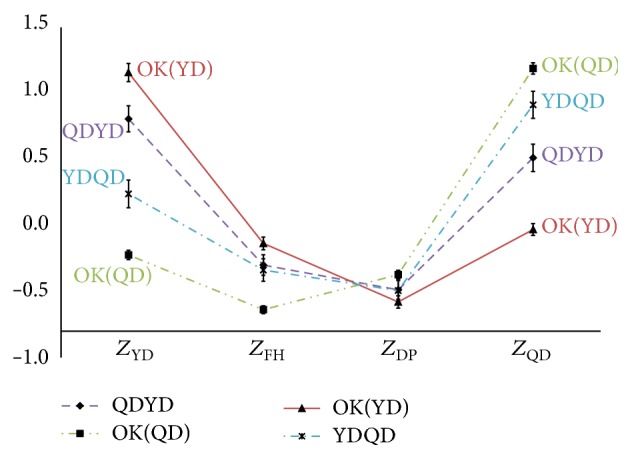
The profiles graphs of the QD and YD. *Z*_FH_: the standardized scores for upper-class variables according to Fire-heat pattern; *Z*_QD_: the standardized scores for upper-class variables according to Qi deficiency pattern; *Z*_DP_: the standardized scores for upper-class variables according to Dampness-phlegm pattern; *Z*_YD_: the standardized scores for upper-class variables according to Yin deficiency pattern; OK: the correct classification types.

**Figure 4 fig4:**
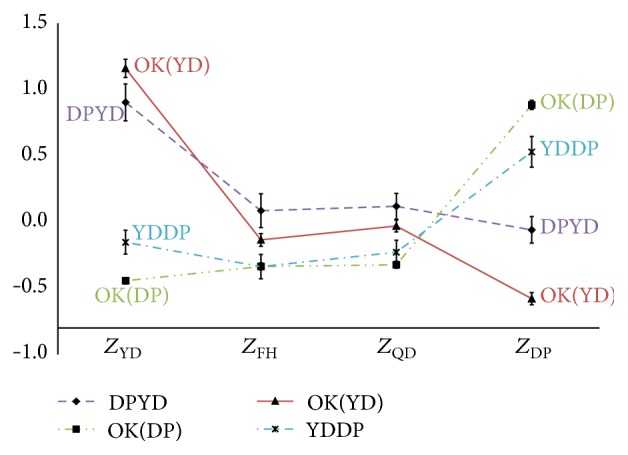
The profiles graphs of the DP and YD. *Z*_FH_: the standardized scores for upper-class variables according to Fire-heat pattern; *Z*_QD_: the standardized scores for upper-class variables according to Qi deficiency pattern; *Z*_DP_: the standardized scores for upper-class variables according to Dampness-phlegm pattern; *Z*_YD_: the standardized scores for upper-class variables according to Yin deficiency pattern; OK: the correct classification types.

**Figure 5 fig5:**
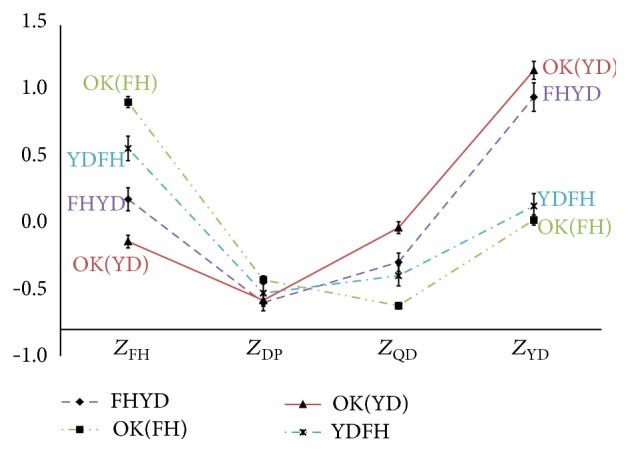
The profiles graphs of the FH and YD. *Z*_FH_: the standardized scores for upper-class variables according to Fire-heat pattern; *Z*_QD_: the standardized scores for upper-class variables according to Qi deficiency pattern; *Z*_DP_: the standardized scores for upper-class variables according to Dampness-phlegm pattern; *Z*_YD_: the standardized scores for upper-class variables according to Yin deficiency pattern; OK: the correct classification types.

**Figure 6 fig6:**
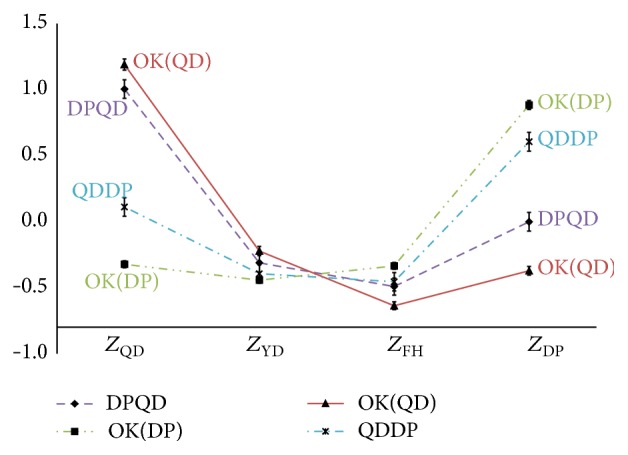
The profiles graphs of the DP and QD. *Z*_FH_: the standardized scores for upper-class variables according to Fire-heat pattern; *Z*_QD_: the standardized scores for upper-class variables according to Qi deficiency pattern; *Z*_DP_: the standardized scores for upper-class variables according to Dampness-phlegm pattern; *Z*_YD_: the standardized scores for upper-class variables according to Yin deficiency pattern; OK: the correct classification types.

**Figure 7:**
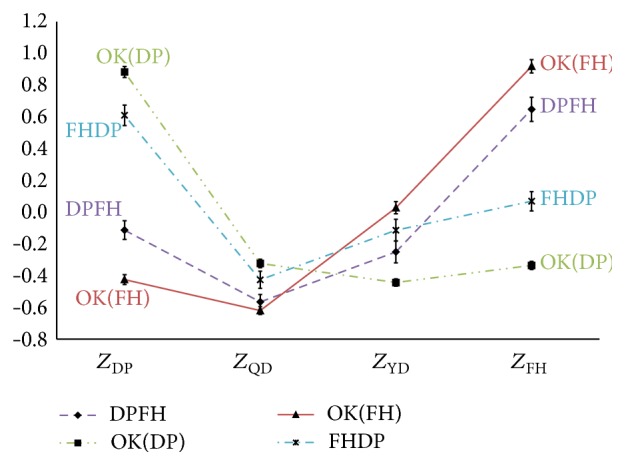
The profiles graphs of the DP and FH. *Z*_FH_: the standardized scores for upper-class variables according to Fire-heat pattern; *Z*_QD_: the standardized scores for upper-class variables according to Qi deficiency pattern; *Z*_DP_: the standardized scores for upper-class variables according to Dampness-phlegm pattern; *Z*_YD_: the standardized scores for upper-class variables according to Yin deficiency pattern; OK: the correct classification types.

With 12 misclassification types and 4 correct classification types categorized by the discriminant analysis, the profile graphs were drawn. Specifically, two of the 4 patterns were selected and the correct classification types and misclassification types for each pattern were collected from the TKM physicians and divided. For instance, as described in Figure 2, patients applicable to two misclassification types (FHQD and QDFH) were grouped together. Next, the upper-class variable scores of each patient were used to draw a profile plot. At this point, it was critical to arrange the pattern scores of correct classification on the edges and those of the other two pattern scores inside. The profile graphs of the misclassification types (FHQD, QDFH, etc.) and the correct classification types (e.g., FH, QD, YD, and DP) are depicted in Figures 2 [Fig fig3] [Fig fig4] [Fig fig5] [Fig fig6]–7 and the relevant statistics are in Table 3. As illustrated in Figures 2–7, two misclassification types demonstrate a U-shaped pattern and correct classification types an L-shaped or flipped-L-shaped pattern.

#### 2.3.3. Derived Four Measures (*D*, *R*, *S*, and *C* Scores)

In the profile graphs, misclassification observations in most of the 6 cases displayed a bathtub or U-shaped pattern since pattern scores corresponding to actual patterns would be relatively high and the misclassification of a pattern is highly probable if relatively higher scores were observed in the other pattern. In the meantime, correct classification observations showed an L-shaped (or flipped-L-shaped) pattern. Although actual patterns are unknown due to the lack of direct diagnoses from TKM physicians, if a new patient establishes a bathtub-shaped profile simply with 4 upper-class pattern scores (obligatory two high scores and two low scores), this patient is likely to be misclassified through the future discriminant model. Criteria were designed to assess how close a pattern score profile would be to a bathtub shape through various arrangements and simple calculations of the four pattern scores and applied to already discriminated data. By doing so, comparison was conducted to investigate how much misclassification was estimated and how much discrimination rates improved when the estimated misclassification observations were eliminated beforehand.

**Figure 8:**
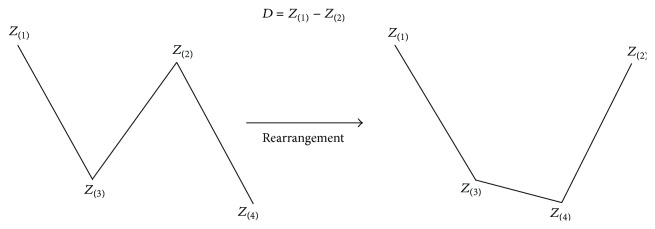
Derived *D* values based on the pattern analysis of the profile graphs. Under the hypothesis that the smaller the *D* value was, the closer the profile graph was to a bathtub (or U) shape, and the higher the probability of the respective observations corresponding to misclassification was.

*(1) D Score*. Analyzing correct classification and misclassification types with profile graphs, the *D* value was derived considering that a difference between the maximum value *Z*_(1)_ and the second-largest value *Z*_(2)_ of misclassification was smaller than that of correct classification, and classification by the value was attempted (Figure 8). Namely, under the hypothesis that the smaller the *D* value was, the closer the profile graph was to a bathtub shape and the higher the probability of the respective observations corresponding to misclassification was, the *D* values were applied to the clinical stroke data.

**Figure 9:**
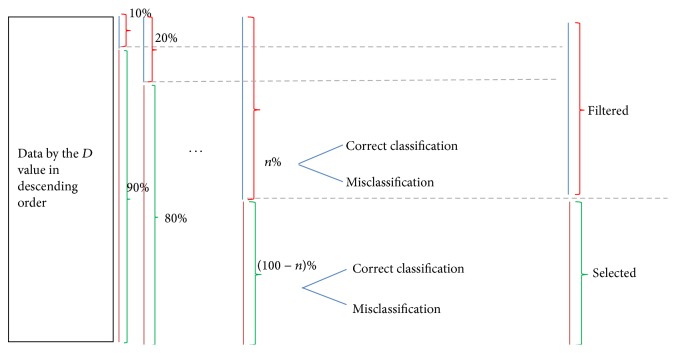
Data filtering and selection method. Data were ranged according to each four measures (*D*, *R*, *S*, and *C* values) in descending or ascending order by increasing data by 10% intervals.

After sorting the data by the *D* value in descending order and investigating the frequency and rates of misclassification over 10% intervals (Figure 9), the misclassification probability of the 10% (*N* = 331) filtered data reached 40.79% (*N*_*m*_ = 135, Mean_*m*_ = 0.058), which was 7.61%  *p* higher than the previously calculated misclassification probability (33.18%) of the total data. The misclassification probabilities of the data filtered from 20% to 90% were lower than that of the 10% filtered data but higher than that of the total data (33.18%). In the data filtered at 10%, 20%, 40%, and 50%, average *D* values of the misclassifications and correct classifications were barely different from each other, even though the average *D* values of the misclassifications tended to be higher than those of the correct classifications. In the other data groups, the average *D* values of the correct classifications were higher than those of the misclassifications (Table 4). Meanwhile, examining the frequencies and rates of the correct classifications in the data selected for *D* values, the misclassification probability of the correct classifications in the 90% (*N* = 2975) selected data recorded 67.66% (*N*_*c*_ = 2013, % of *N*_*m*_ = 32.34%), which was 0.86%  *p* higher than those of the previously calculated correct classifications (66.8%) of the total data. In the 80% (*N* = 2645) selected data, the misclassification probabilities of correct classifications reached 68.28% (*N*_*c*_ = 1806, % of *N*_*m*_ = 31.72%), which was 0.62%  *p* higher than those in the 90% selected data. In the data selected from 70% to 10%, the correct classifications gradually increased (Table 4).

**Figure 10:**
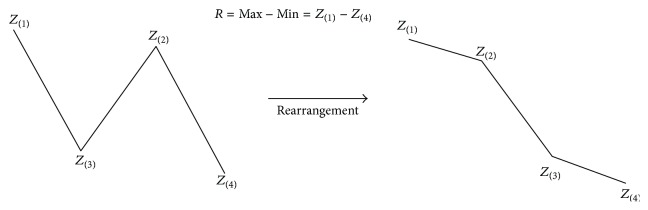
Derived *R* values based on the pattern analysis of the profile graphs. Under the hypothesis that the larger the *R* value was, the closer the profile graph was to an L-shaped or flipped-L-shaped pattern, the higher the probability of the respective observations corresponding to correct classification was.

*(2) R Score*. Analyzing correct classification and misclassification types with profile graphs, the *R* value was derived considering that a difference between the maximum value *Z*_(1)_ and the minimum value *Z*_(4)_ of misclassification was smaller than that of correct classification, and classification by the value was attempted (Figure 10). Namely, under the hypothesis that the larger the *R* value was, the closer the profile graph was to an L-shaped or flipped-L-shaped pattern, and the higher the probability of the respective observations corresponding to correct classification was the *R* values were applied to the clinical stroke data in the same way as previously (Table 5).

**Figure 11:**
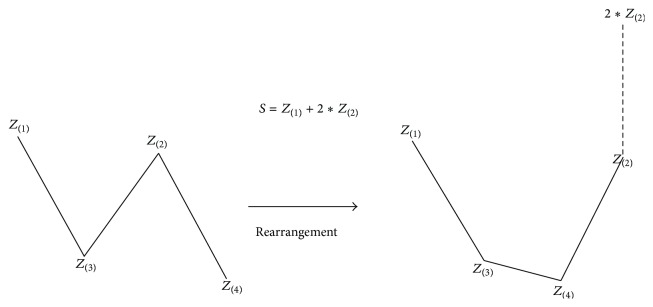
Derived *S* values based on the pattern analysis of the profile graphs. Under the hypothesis that the larger the *S* value was, the closer the profile graph was to a bathtub (or U) shape, the higher the probability of the respective observations corresponding to misclassification was.

*(3) S Score*. Analyzing correct classification and misclassification types with profile graphs, the *S* value was derived considering that the second-largest value *Z*_(2)_ of misclassification was higher than that of correct classification, and classification by the value was attempted (Figure 11). Namely, under the hypothesis that the larger the *S* value was, the closer the profile graph was to a bathtub (or U) shape and the higher the probability of the respective observations corresponding to misclassification was, the *S* values were applied to the clinical stroke data. In this case, the frequency and rates of misclassification over 10% intervals were investigated after sorting the data by the *S* value in ascending order (Table 6).

**Figure 12:**
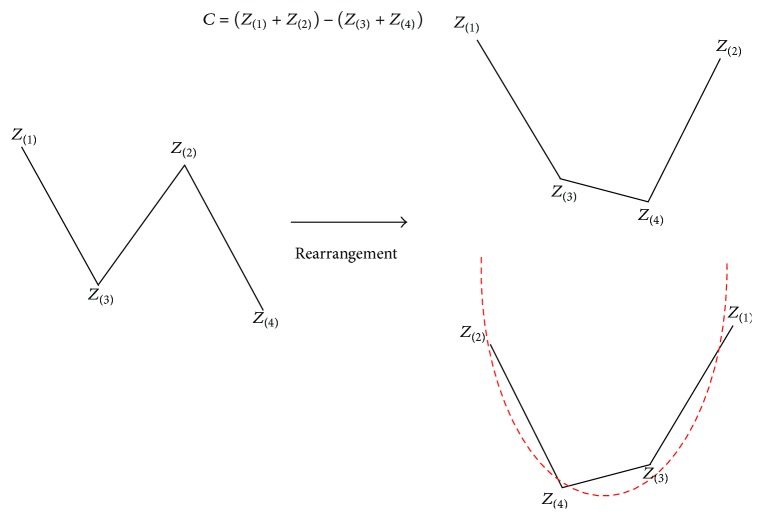
Derived *C* values based on the pattern analysis of the profile graphs. Under the hypothesis that the larger the *C* value was, the closer the profile graph was to a bathtub (or U) shape, the higher the probability of the respective observations corresponding to misclassification was.

*(4) C Score*. Analyzing correct classification and misclassification types with profile graphs, the *C* value was derived considering that a difference between the sum of *Z*_(1)_ and *Z*_(2)_ and the sum of *Z*_(3)_ and *Z*_(4)_ of misclassification was larger than that of correct classification, and classification by the value was attempted (Figure 12). Namely, under the hypothesis that the larger the *C* value was, the closer the profile graph was to a bathtub (or U) shape, the higher the probability of the respective observations corresponding to misclassification was, the *C* values were applied to the clinical stroke data in the same way as previously (Table 7).

## 3. Results

### 3.1. Estimated Misclassification Probability and Discrimination Rate according to Proposed Four Scores

Table 8 summarizes the misclassification probabilities after the data was sorted according to the 4 criteria and investigating the misclassification probability over 10% intervals. If the data were filtered 10–20%, the *C* score marked 42.60% and 41.15%, respectively, indicating the highest misclassification probability among the criteria. If the data were filtered 30%, the *R* score stands at 40.32% and the *C* score at 39.92%. If the data were filtered 40~90%, the misclassification probability of the *D* score was the highest.

For the data previously selected by 4 scores (*D*, *R*, *S*, and *C*), discrimination rates were compared. Having the 4 QD, DP, YD, and FH patterns set as reaction variables for the entire clinical stroke data and 44 clinical indices of the* Korean Standard PI for Stroke-3* as independent variables, the discriminant analysis was conducted to calculate the discrimination accuracy (Table 9). If the data were selected at 90%, the discrimination rate of the *D* score increased to 68.2%, which was the largest increase among the four scores. If the data were selected at 80%, the *C* score reached 69.0%, making the largest increase. If the data were selected at 70%, the *R* score posted 70.0%, demonstrating the largest increase in the discrimination rate among the four scores. If the data were selected at 60–10%, the *D* score recorded the largest increase in the discrimination rate among the four scores.

### 3.2. Similarities between Secondary Curvature Function and C Score

#### 3.2.1. Curvature Created by *Z*_(1)_, *Z*_(2)_, *Z*_(3)_, and *Z*_(4)_ Scores

**Figure 13:**
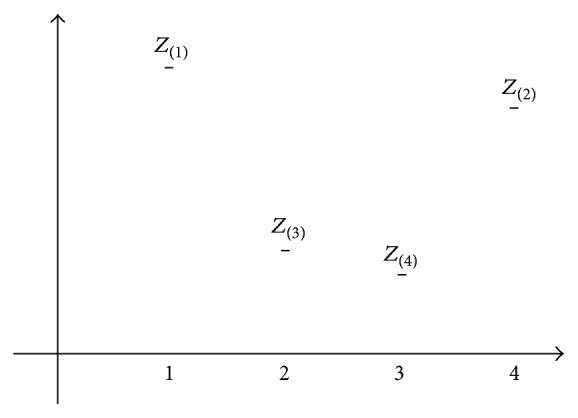
Curvature created by *Z* scores (*Z*_(1)_, *Z*_(2)_, *Z*_(3)_, and *Z*_(4)_). *Z*_(1)_, *Z*_(2)_, *Z*_(3)_, and *Z*_(4)_, as dependent variables observed in the *x* values having equal intervals. *Z*_(1)_ is a dependent variable when *x* = 1, *Z*_(2)_ when *x* = 4, *Z*_(3)_ when *x* = 2, and *Z*_(4)_ when *x* = 3.

First of all, assume four scores, *Z*_(1)_, *Z*_(2)_, *Z*_(3)_, and *Z*_(4)_, as dependent variables observed in the *x* values (e.g., 1, 2, 3, and 4) having equal intervals, as shown in the profile graphs. In addition, assume that *Z*_(1)_ is a dependent variable when *x* = 1, *Z*_(2)_ when *x* = 4, *Z*_(3)_ when *x* = 2, and *Z*_(4)_ when *x* = 3. This assumption is illustrated in Figure 13.

#### 3.2.2. Estimation of Secondary Curvature

Considering the quadratic curve regression model passing through the four points (1, *Z*_(1)_), (2, *Z*_(3)_), (3, *Z*_(4)_), and (4, *Z*_(2)_), *Y* = *β*_0_ + *β*_1_ *X* + *β*_2_ *X* ^2^ + *ϵ*, the coefficient of *β*_2_ is the secondary curvature value that we wanted. Namely, the larger the *β*_2_ is, the stronger the bathtub shape becomes, boosting the misclassification probability. Assuming that the estimates of *β*_0_, *β*_1_, and *β*_2_ are *b*_0_, *b*_1_, and *b*_2_, these estimates satisfy the following normal equation [21]:

(1) $$\begin{array}{c} \left(\;X^{\prime }X\;\right)b=X^{\prime }Y. \end{array}$$

Here

(2) X=1112122213321442,b=b0b1b2,Y=Z1Z2Z3Z4.

According to Neter et al. [21], a general two-variable regression model,

(3) $$\begin{array}{c} Y_{i}=\beta_{0}+\beta_{1}X_{i1}+\beta_{2}X_{i2}+\epsilon_{i}, \end{array}$$

has a normal equation

(4) $$\begin{array}{c} \left(\;X^{\prime }X\;\right)b=X^{\prime }Y, \end{array}$$

which is equal to

(5) $$\begin{array}{c} \left[\begin{array}{ccc} n & \sum X_{i1} & \sum X_{i2}\\ \sum X_{i1} & \sum X_{i1}^{2} & \sum X_{i1}X_{i2}\\ \sum X_{i2} & \sum X_{i2}X_{i1} & \sum {X^{2}}_{i2} \end{array}\right]\left[\begin{array}{c} b_{0}\\ b_{1}\\ b_{2} \end{array}\right]=\left[\begin{array}{c} \sum Y_{i}\\ \sum {X_{i1}Y}_{i}\\ \sum {X_{i2}Y}_{i} \end{array}\right], \end{array}$$

and the following normal equations,

(6) ∑Yi=nb0+b1∑Xi1+b2∑Xi2,∑Xi1Yi=b0∑Xi1+b1∑Xi12+b2∑Xi1Xi2∑Xi2Yi=b0∑Xi2+b1∑Xi1Xi2+b2∑Xi22,

are obtained. In this case, the equations are

(7) Xi1=i,i=1,2,3,4,Xi2=i2,i=1,2,3,4,Y1=Z1,Y2=Z3,Y3=Z4,Y4=Z2.

Now, if

(8) S1=Z1+Z2+Z3+Z4,S2=Z1+2Z3+3Z4+4Z2,S3=Z1+4Z3+9Z4+16Z2,

the normal equations should be equal to

(9) S1=4b0+10b1+30b2,S2=10b0+30b1+100b2,S3=30b0+100b1+354b2

and, ultimately, we obtain

(10) ∴b254S1−S2+S35=14Z1+Z2−Z3+Z4.

Certainly, the values of *b*_0_ and *b*_1_ may be obtained but omitted herein because they are meaningless. In (10), *Z*_(1)_ and *Z*_(2)_ are symmetric, and so are *Z*_(3)_ and *Z*_(4)_. Namely, when the curvature creates the largest profile with the 4 points, the curvature will not have any changes even if the largest and the second largest scores were switched. This also holds true for the smallest and the second smallest scores.

In the meantime, the *b*_2_ value equals 1/4 of the *C* score among the 4 criteria obtained. Namely, the previously used *C* score was equal to *Z*_(3)_ and *Z*_(4)_ was simply subtracted from the total of *Z*_(1)_ and *Z*_(2)_, which was the same as the secondary curvature created by the 4 scores.

## 4. Discussion

In TKM, a PI diagnostic system—one of the core technologies in the diagnosis and treatment of oriental medicine—is used to determine the cause and nature of a disease, treatment methods, and treatment drugs for the patients [5–7]. However, the PI diagnosis holds limited objectivity and reproducibility due to the lack of standardized measurement indices. Objectification problems have always arisen with respect to personal deviations among TKM physicians. As the demand for the reestablishment and development of TKM has increased, studies on the establishment of a scientific basis for and the standardization of PI have been actively conducted [7, 12].

In this study, the clinical data of PI diagnosis for stroke were used to analyze and quantify the profile patterns of the misclassification types by applying the proposed scores to the comparative analysis. This was intended to boost the correct classification of objects by detecting those objects with a high probability of actual misclassification and deferring discrimination. Misclassification types were discerned by a discriminant analysis on the actual clinical data of PI diagnosis for stroke and quantified by a profile pattern analysis. The proposed criteria of each standard were applied to the data already discriminated by the previous discriminant analysis in order to compare how well the misclassification had been estimated and how much the discrimination rate had improved when the estimated misclassification observations were removed in advanced. Particularly, the *C* score delivered the same results as those from the discrimination of misclassification observations through a secondary curvature. Going forward, the following studies must be performed. First of all, 4 criteria to estimate misclassification were proposed in this study and applied to the actual clinical data, producing the possibility of better estimation of partial misclassification. Nonetheless, it was difficult to notably enhance discrimination rates and additional research appears to be necessary. In addition, 4 pattern groups with a different sample size were used in this study. Hence, the effects of different sample sizes need to be investigated.

## Supplementary Material

This summary is the Korean Standard PI for Stroke-3. It consists of 44 clinical indices and each clinical index belongs to its respective PI types (the Fire-Heat pattern, Yin-Deficiency pattern, Qi-Deficiency pattern, and Dampness-Phlegm pattern).

## Figures and Tables

**Table 1:** Results using the classification of discriminant model.

		Classification result *N* (%)				
		QD	DP	YD	FH	Total
Physician's diagnosis	QD	498 (66.94)	115 (15.46)	95 (12.77)	36 (4.84)	744 (22.50)
	DP	118 (10.61)	783 (70.41)	69 (6.21)	142 (12.77)	1112 (33.64)
	YD	70 (14.64)	55 (11.51)	276 (57.74)	77 (16.11)	478 (14.46)
	FH	46 (4.73)	147 (15.12)	127 (13.07)	652 (67.08)	972 (29.40)
	Total	732 (22.14)	1100 (33.27)	567 (17.15)	907 (27.44)	3306 (100.00)

QD: Qi deficiency pattern; DP: Dampness-phlegm pattern; YD: Yin deficiency pattern; FH: Fire-heat pattern.

**Table 2:** The mean values of the standardized scores for upper-class variables according to misclassification type.

Types of misclassification		*N* (%)	*Z*_QD_	*Z*_DP_	*Z*_YD_	*Z*_FH_
1	DPFH^#^	142 (12.94)	−0.565	−0.113	−0.251	0.648
2	DPQD	118 (10.76)	1.004	−0.001	−0.312	−0.492
3	DPYD	69 (6.29)	0.118	−0.060	0.902	0.085
4	FHDP	147 (13.40)	−0.426	0.610	−0.114	0.069
5	FHQD	46 (4.19)	0.907	−0.494	−0.233	0.096
6	FHYD	127 (11.58)	−0.291	−0.596	0.956	0.184
7	QDDP	115 (10.48)	0.111	0.605	−0.394	−0.456
8	QDFH	36 (3.28)	0.075	−0.500	−0.373	0.560
9	QDYD	95 (8.66)	0.512	−0.487	0.808	−0.299
10	YDDP	55 (5.01)	−0.229	0.529	−0.153	−0.336
11	YDFH	77 (7.02)	−0.393	−0.525	0.133	0.568
12	YDQD	70 (6.38)	0.914	−0.492	0.240	−0.337

Total		1097 (100.00)	0.067	−0.063	0.110	0.017

QD: Qi deficiency pattern; DP: Dampness-phlegm pattern; YD: Yin deficiency pattern; FH: Fire-heat pattern; DPFH^#^: physician's diagnosis- Dampness-phlegm pattern, classification result, Fire-heat pattern; *Z*_QD_: the standardized scores for upper-class variables according to Qi deficiency pattern; *Z*_DP_: the standardized scores for upper-class variables according to Dampness-phlegm pattern; *Z*_YD_: the standardized scores for upper-class variables according to Yin deficiency pattern; *Z*_FH_: the standardized scores for upper-class variables according to Fire-heat pattern.

**Table 3:** Summary of *Z* scores according to the profile graphs for PI classification types.

	Classification types	*N*	*Z* scores (mean ± SE)			
			*Z*_QD_	*Z*_DP_	*Z*_YD_	*Z*_FH_
FH, QD classification types	FHQD	46	0.907 ± 0.137	−0.494 ± 0.110	−0.233 ± 0.109	0.097 ± 0.120
	OK(FH)	652	−0.620 ± 0.025	−0.425 ± 0.031	0.028 ± 0.038	0.919 ± 0.042
	OK(QD)	498	1.189 ± 0.043	−0.372 ± 0.033	−0.223 ± 0.035	−0.637 ± 0.030
	QDFH	36	0.075 ± 0.130	−0.500 ± 0.107	−0.373 ± 0.118	0.560 ± 0.175
	Total	1232	0.189 ± 0.034	−0.408 ± 0.022	−0.095 ± 0.025	0.249 ± 0.034

QD, YD classification types	QDYD	95	0.513 ± 0.103	−0.487 ± 0.072	0.808 ± 0.099	−0.300 ± 0.078
	OK(QD)	498	1.189 ± 0.043	−0.372 ± 0.033	−0.223 ± 0.035	−0.637 ± 0.030
	OK(YD)	276	−0.031 ± 0.045	−0.579 ± 0.046	1.159 ± 0.068	−0.135 ± 0.048
	YDQD	70	0.914 ± 0.102	−0.493 ± 0.090	0.240 ± 0.105	−0.337 ± 0.085
	Total	939	0.742 ± 0.034	−0.454 ± 0.024	0.322 ± 0.036	−0.433 ± 0.025

DP, YD classification types	DPYD	69	0.118 ± 0.097	−0.060 ± 0.101	0.903 ± 0.139	0.085 ± 0.127
	OK(DP)	783	−0.323 ± 0.027	0.883 ± 0.034	−0.443 ± 0.024	−0.336 ± 0.026
	OK(YD)	276	−0.031 ± 0.045	−0.579 ± 0.046	1.159 ± 0.068	−0.135 ± 0.048
	YDDP	55	−0.229 ± 0.090	0.529 ± 0.116	−0.153 ± 0.090	−0.336 ± 0.092
	Total	1183	−0.225 ± 0.022	0.471 ± 0.032	0.022 ± 0.032	−0.264 ± 0.023

FH, YD classification types	FHYD	127	−0.291 ± 0.069	−0.597 ± 0.063	0.956 ± 0.108	0.184 ± 0.087
	OK(FH)	652	−0.620 ± 0.025	−0.425 ± 0.031	0.028 ± 0.038	0.919 ± 0.042
	OK(YD)	276	−0.031 ± 0.045	−0.579 ± 0.046	1.159 ± 0.068	−0.135 ± 0.048
	YDFH	77	−0.393 ± 0.077	−0.525 ± 0.086	0.133 ± 0.095	0.568 ± 0.093
	Total	1132	−0.424 ± 0.022	−0.489 ± 0.023	0.415 ± 0.034	0.555 ± 0.032

DP, QD classification types	DPQD	118	1.004 ± 0.071	−0.001 ± 0.071	−0.312 ± 0.070	−0.492 ± 0.064
	OK(DP)	783	−0.323 ± 0.027	0.883 ± 0.034	−0.443 ± 0.024	−0.336 ± 0.026
	OK(QD)	498	1.189 ± 0.043	−0.372 ± 0.033	−0.223 ± 0.035	−0.637 ± 0.030
	QDDP	115	0.111 ± 0.070	0.605 ± 0.071	−0.395 ± 0.067	−0.456 ± 0.069
	Total	1514	0.311 ± 0.028	0.380 ± 0.027	−0.357 ± 0.019	−0.456 ± 0.018

DP, FH classification types	DPFH	142	−0.565 ± 0.047	−0.113 ± 0.059	−0.251 ± 0.069	0.648 ± 0.076
	OK(DP)	783	−0.323 ± 0.027	0.883 ± 0.034	−0.443 ± 0.024	−0.336 ± 0.026
	OK(FH)	652	−0.620 ± 0.025	−0.425 ± 0.031	0.028 ± 0.038	0.919 ± 0.042
	FHDP	147	−0.426 ± 0.054	0.610 ± 0.064	−0.114 ± 0.068	0.069 ± 0.061
	Total	1724	−0.464 ± 0.017	0.283 ± 0.026	−0.221 ± 0.020	0.254 ± 0.026

PI: pattern identification; QD: Qi deficiency pattern; DP: Dampness-phlegm pattern; YD: Yin deficiency pattern; FH: Fire-heat pattern; OK: the correct classification types; *Z*_QD_: the standardized scores for upper-class variables according to Qi deficiency pattern; *Z*_DP_: the standardized scores for upper-class variables according to Dampness-phlegm pattern; *Z*_YD_: the standardized scores for upper-class variables according to Yin deficiency pattern; *Z*_FH_: the standardized scores for upper-class variables according to Fire-heat pattern.

**Table 4:** Types of classifications distribution of filtered/selected data by *D* value.

Filtered%	Type of classifications distribution of filtered data by *D* value						Selected%	Type of classifications distribution of selected data by *D* value					
	*N*_*m*_ (%)	*N*_*c*_ (%)	*N*_*t*_ (%)	Mean_*m*_	Mean_*c*_	Mean_*t*_		*N*_*m*_ (%)	*N*_*c*_ (%)	*N*_*t*_ (%)	Mean_*m*_	Mean_*c*_	Mean_*t*_
10%	135 (40.79)	196 (59.21)	331 (100)	0.058	0.053	0.055	10%	42 (12.69)	289 (87.31)	331 (100)	2.399	2.531	2.515
20%	258 (39.03)	403 (60.97)	661 (100)	0.124	0.125	0.125	20%	119 (18.00)	542 (82.00)	661 (100)	1.913	2.112	2.076
30%	382 (38.51)	610 (61.49)	992 (100)	0.184	0.184	0.184	30%	232 (23.39)	760 (76.61)	992 (100)	1.585	1.870	1.804
40%	525 (39.71)	797 (60.29)	1322 (100)	0.252	0.242	0.246	40%	338 (25.57)	984 (74.43)	1322 (100)	1.397	1.668	1.599
50%	647 (39.14)	1006 (60.86)	1653 (100)	0.319	0.319	0.319	50%	450 (27.22)	1203 (72.78)	1653 (100)	1.244	1.508	1.436
60%	759 (38.26)	1225 (61.74)	1984 (100)	0.387	0.402	0.396	60%	572 (28.83)	1412 (71.17)	1984 (100)	1.107	1.375	1.298
70%	865 (37.38)	1449 (62.62)	2314 (100)	0.460	0.492	0.480	70%	715 (30.90)	1599 (69.10)	2314 (100)	0.973	1.265	1.175
80%	978 (36.98)	1667 (63.02)	2645 (100)	0.550	0.593	0.578	80%	839 (31.72)	1806 (68.28)	2645 (100)	0.875	1.154	1.065
90%	1055 (35.46)	1920 (64.54)	2975 (100)	0.630	0.731	0.695	90%	962 (32.34)	2013 (67.66)	2975 (100)	0.788	1.055	0.969

*N*_*m*_: number of misclassification types; *N*_*c*_: number of correct classification types; *N*_*t*_: number of total classification types; Mean_*m*_: mean of misclassification type; Mean_*c*_: mean of correct classification type; Mean_*t*_: mean of total classification type.

**Table 5:** Types of classifications distribution of filtered/selected data by *R* value.

Filtered%	Type of classifications distribution of filtered data by *R* value						Selected%	Type of classifications distribution of selected data by *R* value					
	*N*_*m*_ (%)	*N*_*c*_ (%)	*N*_*t*_ (%)	Mean_*m*_	Mean_*c*_	Mean_*t*_		*N*_*m*_ (%)	*N*_*c*_ (%)	*N*_*t*_ (%)	Mean_*m*_	Mean_*c*_	Mean_*t*_
10%	135 (40.79)	196 (59.21)	331 (100)	0.674	0.677	0.676	10%	65 (19.64)	266 (80.36)	331 (100)	3.790	3.882	3.864
20%	261 (39.49)	400 (60.51)	661 (100)	0.847	0.864	0.858	20%	160 (24.21)	501 (75.79)	661 (100)	3.247	3.418	3.376
30%	400 (40.32)	592 (59.68)	992 (100)	0.991	0.990	0.990	30%	254 (25.60)	738 (74.40)	992 (100)	2.967	3.116	3.078
40%	507 (38.35)	815 (61.65)	1322 (100)	1.099	1.130	1.118	40%	371 (28.06)	951 (71.94)	1322 (100)	2.719	2.905	2.853
50%	623 (37.69)	1030 (62.31)	1653 (100)	1.212	1.252	1.234	50%	474 (28.68)	1179 (71.32)	1653 (100)	2.542	2.710	2.662
60%	726 (36.59)	1258 (63.41)	1984 (100)	1.310	1.369	1.347	60%	590 (29.74)	1394 (70.26)	1984 (100)	2.377	2.556	2.503
70%	843 (36.43)	1471 (63.57)	2314 (100)	1.431	1.486	1.466	70%	697 (30.12)	1617 (69.88)	2314 (100)	2.243	2.411	2.360
80%	937 (35.43)	1708 (64.57)	2645 (100)	1.537	1.623	1.593	80%	836 (31.61)	1809 (68.39)	2645 (100)	2.080	2.288	2.222
90%	1032 (34.69)	1943 (65.31)	2975 (100)	1.660	1.777	1.736	90%	962 (32.34)	2013 (67.66)	2975 (100)	1.942	2.162	2.091

*N*_*m*_: number of misclassification types; *N*_*c*_: number of correct classification types; *N*_*t*_: number of total classification types; Mean_*m*_: mean of misclassification types; Mean_*c*_: mean of correct classification types; Mean_*t*_: mean of total classification types.

**Table 6:** Types of classifications distribution of filtered/selected data by *S* value.

Filtered%	Type of classifications distribution of filtered data by *S* value						Selected%	Type of classifications distribution of selected data by *S* value					
	*N*_*m*_ (%)	*N*_*c*_ (%)	*N*_*t*_ (%)	Mean_*m*_	Mean_*c*_	Mean_*t*_		*N*_*m*_ (%)	*N*_*c*_ (%)	*N*_*t*_ (%)	Mean_*m*_	Mean_*c*_	Mean_*t*_
10%	120 (36.25)	211 (63.75)	331 (100)	5.587	5.763	5.699	10%	100 (30.21)	231 (69.79)	331 (100)	−1.678	−1.625	−1.641
20%	234 (35.40)	427 (64.60)	661 (100)	4.620	4.673	4.654	20%	205 (31.01)	456 (68.99)	661 (100)	−1.159	−1.162	−1.161
30%	333 (33.57)	659 (66.43)	992 (100)	4.051	3.975	4.000	30%	312 (31.45)	680 (68.55)	992 (100)	−0.792	−0.804	−0.800
40%	435 (32.90)	887 (67.10)	1322 (100)	3.580	3.475	3.509	40%	431 (32.60)	891 (67.40)	1322 (100)	−0.469	−0.516	−0.501
50%	554 (33.51)	1099 (66.49)	1653 (100)	3.126	3.085	3.099	50%	543 (32.85)	1110 (67.15)	1653 (100)	−0.167	−0.226	−0.207
60%	666 (33.57)	1318 (66.43)	1984 (100)	2.768	2.731	2.743	60%	662 (33.37)	1322 (66.63)	1984 (100)	0.127	0.043	0.071
70%	785 (33.92)	1529 (66.08)	2314 (100)	2.405	2.411	2.409	70%	764 (33.02)	1550 (66.98)	2314 (100)	0.382	0.335	0.351
80%	892 (33.72)	1753 (66.28)	2645 (100)	2.106	2.093	2.097	80%	863 (32.63)	1782 (67.37)	2645 (100)	0.649	0.642	0.644
90%	997 (33.51)	1978 (66.49)	2975 (100)	1.814	1.777	1.789	90%	977 (32.84)	1998 (67.16)	2975 (100)	0.993	0.963	0.973

*N*_*m*_: number of misclassification types; *N*_*c*_: number of correct classification types; *N*_*t*_: number of total classification types; Mean_*m*_: mean of misclassification type; Mean_*c*_: mean of correct classification type; Mean_*t*_: mean of total classification type.

**Table 7:** Types of classifications distribution of filtered/selected data by *C* value.

Filtered%	Type of classifications distribution of filtered data by *C* value						Selected%	Type of classifications distribution of selected data by *C* value					
	*N*_*m*_ (%)	*N*_*c*_ (%)	*N*_*t*_ (%)	Mean_*m*_	Mean_*c*_	Mean_*t*_		*N*_*m*_ (%)	*N*_*c*_ (%)	*N*_*t*_ (%)	Mean_*m*_	Mean_*c*_	Mean_*t*_
10%	141 (42.60)	190 (57.40)	331 (100)	0.846	0.845	0.845	10%	84 (25.38)	247 (74.62)	331 (100)	5.037	5.134	5.110
20%	272 (41.15)	389 (58.85)	661 (100)	1.066	1.085	1.078	20%	177 (26.78)	484 (73.22)	661 (100)	4.345	4.463	4.431
30%	396 (39.92)	596 (60.08)	992 (100)	1.240	1.267	1.256	30%	273 (27.52)	719 (72.48)	992 (100)	3.928	4.040	4.009
40%	516 (39.03)	806 (60.97)	1322 (100)	1.392	1.426	1.413	40%	370 (27.99)	952 (72.01)	1322 (100)	3.636	3.737	3.708
50%	619 (37.45)	1034 (62.55)	1653 (100)	1.520	1.588	1.562	50%	478 (28.92)	1175 (71.08)	1653 (100)	3.378	3.498	3.463
60%	727 (36.64)	1257 (63.36)	1984 (100)	1.664	1.746	1.716	60%	581 (29.28)	1403 (70.72)	1984 (100)	3.161	3.281	3.246
70%	824 (35.61)	1490 (64.39)	2314 (100)	1.799	1.911	1.871	70%	701 (30.29)	1613 (69.71)	2314 (100)	2.945	3.098	3.051
80%	920 (34.78)	1725 (65.22)	2645 (100)	1.941	2.082	2.033	80%	825 (31.19)	1820 (68.81)	2645 (100)	2.746	2.928	2.871
90%	1013 (34.05)	1962 (65.95)	2975 (100)	2.105	2.285	2.224	90%	956 (32.13)	2019 (67.87)	2975 (100)	2.548	2.769	2.698

*N*_*m*_: number of misclassification types; *N*_*c*_: number of correct classification types; *N*_*t*_: number of total classification types; Mean_*m*_: mean of misclassification type; Mean_*c*_: mean of correct classification type; Mean_*t*_: mean of total classification type.

**Table 8:** Misclassification rate distribution of the filtered data according to four measures.

Filtered%	*N*	*D*	*R*	*S*	*C*
10%	331	40.79	40.79	36.25	42.60
20%	661	39.03	39.49	35.40	41.15
30%	992	38.51	40.32	33.57	39.92
40%	1322	39.71	38.35	32.90	39.03
50%	1653	39.14	37.69	33.51	37.45
60%	1984	38.26	36.59	33.57	36.64
70%	2314	37.38	36.43	33.92	35.61
80%	2645	36.98	35.43	33.72	34.78
90%	2975	35.46	34.69	33.51	34.05

**Table 9:** Discriminant rate distribution of the selected data according to four measures.

	Discriminant rate				
	*N*	*D*	*R*	*S*	*C*
100%	3306	66.82	66.82	66.82	66.82
90%	2975	**68.24 (+1.42)**	67.63 (+0.81)	66.92 (+0.10)	67.53 (+0.71)
80%	2645	68.62 (+0.38)	68.47 (+0.84)	67.15 (+0.23)	**69.04 (+1.51)**
70%	2314	69.53 (+0.91)	**69.97 (+1.50)**	66.98 (−0.17)	69.49 (+0.45)
60%	1984	**71.98 (+2.45)**	70.82 (+0.85)	66.94 (−0.04)	71.22 (+1.73)
50%	1653	**73.32 (+1.34)**	73.08 (+2.26)	69.03 (+2.09)	71.81 (+0.59)
40%	1322	**75.34 (+2.02)**	74.28 (+1.20)	68.68 (−0.35)	73.75 (+1.94)
30%	992	**77.32 (+1.98)**	76.81 (+2.53)	70.26 (+1.58)	75.81 (+2.06)
20%	661	**83.36 (+6.04)**	80.94 (+4.13)	73.83 (+3.57)	77.61 (+1.80)
10%	331	**89.12 (+5.76)**	87.01 (+6.07)	75.83 (+2.00)	82.78 (+5.17)
