# Supplementary material for: The Study of Misclassification Probability in Discriminant Model of Pattern Identification for Stroke
Source: Evid Based Complement Alternat Med. 2016 Mar 10;2016:1912897. doi: 10.1155/2016/1912897 (PMC4806281; doi:10.1155/2016/1912897)
Supplement: Supplementary file 1 — This summary is the Korean Standard PI for Stroke-3. It consists of 44 clinical indices and each clinical index belongs to its respective PI types (the Fire-Heat pattern, Yin-Deficiency pattern, Qi-Deficiency pattern, and Dampness-Phlegm pattern). [file 1912897.f1.pdf]

Supplemental table 1. Korean Standard PI for Stroke-3

Fire-heat pattern (19 variable)	Yin deficiency pattern (7 variable)	Qi deficiency pattern (11 variable)	Dampness-phlegm pattern (7 variable)
1. Reddened complexion	1. Pale face and red zygomatic-site	1. Pale complexion	1. Sallow complexion
2. Headache like flush	2. Tidal fever	2. Looks powerless and lazy	2. Heavy
3. Heat vexation and aversion to heat	3. Thin	3. Feels powerless and lazy	3. Dark inferior palpebral
4. Heat vexation in the chest	4. Night sweating	4. Reluctance to speak	4. Dizziness with nausea
5. Vexation and insomnia	5. Dry mouth	5. Drowsiness, likes to lie down	5. (tongue) Enlarged tongue
6. Thirst	6. (tongue) Bare and red tongue like a mirror	6. Reversed cold in the extremities	6. (tongue) White fur on tongue
7. Wheezing in the throat with sputum	7. (tongue) Dry fur on tongue	7. (tongue) Pale tongue	7. (pulse) Slippery pulse
8. Blood-shot eyes		8. (tongue) Teeth-marked tongue	
9. Aphtha or tongue sore		9. (pulse) Weak pulse	
10. Heat in the palms and soles		10. (pulse) Fine pulse	
11. Vexing heat in the extremities		11. (pulse) Slow pulse	
12. Turbid urine			
13. Fetid mouth odor			
14. (tongue) Yellow fur			
15. (tongue) Thick fur			
16. (tongue) Red tongue			
17. (pulse) Strong pulse			
18. (pulse) Surging pulse			
19. (pulse) Rapid pulse			

PI, pattern identification;
